# Prognostic Value of Intraoperative Distal Radioulnar Joint Instability Grading in Distal Radius and Galeazzi Fractures: A Prospective Multicenter Cohort Study

**DOI:** 10.3390/life16030437

**Published:** 2026-03-09

**Authors:** Awad Dmour, Yousef Khair, Almuthanna Alyamani, Paul-Dan Sirbu, Bianca-Ana Dmour, Ahmad Al-Zoubi, Yousef Al-Saraireh, Teodor-Stefan Gheorghevici, Stefan-Dragos Tirnovanu, Dragos-Cristian Popescu, Mihaela Pertea, Alexandra Burlui, Hussein Dmour, Bogdan Puha

**Affiliations:** 1 Grigore T. Popa University of Medicine and Pharmacy Iasi, 700115 Iasi, Romania; dmour-awad@umfiasi.ro (A.D.); bianca-ana.dmour@umfiasi.ro (B.-A.D.); dragos.popescu@umfiasi.ro (D.-C.P.); mihaela.pertea@umfiasi.ro (M.P.); alexandra.burlui@umfiasi.ro (A.B.); bogdan.puha@umfiasi.ro (B.P.); 2 Orthopaedic and Traumatology Clinic, “Sf. Spiridon” Clinical Emergency Hospital, 700111 Iasi, Romania; steoevici@gmail.com; 3 Royal Medical Services, Amman 11855, Jordan; yousefkh78@yahoo.com (Y.K.); yamaniortho@hotmail.com (A.A.); ahmadz78z@hotmail.com (A.A.-Z.); drdmour65@gmail.com (H.D.); 4 Department of Hand and Upper Limb Surgery, King Hussein Medical Center, King Abdullah II St. 230, Amman 11855, Jordan; 5 Orthopaedic and Sports Surgery, Queen Alia Military Hospital, Jordanian Royal Medical Services, Amman 11855, Jordan; 6 Department of Pharmacology, Faculty of Medicine, Mutah University, P.O. Box 7, Al-Karak 61710, Jordan; yousef.sar@mutah.edu.jo

**Keywords:** distal radioulnar joint, intraoperative instability, distal radius fracture, Galeazzi fracture, prognostic classification, clinical validation, functional outcome, QuickDASH, multicenter cohort study

## Abstract

Despite anatomically successful fixation of distal radius and Galeazzi fractures, a subset of patients develops persistent pain and functional limitation, suggesting that factors beyond osseous alignment influence recovery. Distal radioulnar joint instability has been implicated in unfavorable outcomes, yet intraoperative assessment remains inconsistently standardized and has rarely been validated as a prognostic variable. This prospective multicenter observational cohort study included 120 consecutive patients with distal radius or Galeazzi fractures treated with plate fixation in two tertiary centers. After fracture reduction and stabilization, intraoperative distal radioulnar joint stability was systematically assessed using a previously published classification system comprising Grades I to III, with patients demonstrating no instability serving as the reference group. The primary outcome was the QuickDASH score at 12 months, while secondary outcomes included pain intensity, grip strength, radiographic distal radioulnar joint gap, and postoperative complications. Multivariable linear regression was used to evaluate the association between intraoperative instability grade and outcomes, adjusting for age, sex, fracture type, and treatment center. Increasing instability grade was independently associated with worse functional outcome, higher pain levels, reduced grip strength, and greater postoperative distal radioulnar joint widening at 12 months, with an adjusted increase of approximately 5 to 6 QuickDASH points per grade. Intraoperative distal radioulnar joint instability grading provides clinically relevant prognostic information and supports postoperative risk stratification following distal radius and Galeazzi fractures.

## 1. Introduction

Distal radius and Galeazzi fractures are among the most common upper-limb injuries treated surgically. Although modern plate fixation techniques allow reliable restoration of osseous anatomy, a proportion of patients continue to experience persistent pain, reduced grip strength, and functional limitation despite radiographically satisfactory fracture healing. These findings indicate that factors beyond bony alignment influence long-term wrist function [1,2].

Distal radioulnar joint instability has been increasingly recognized as a contributor to unfavorable outcomes following these fractures. While postoperative assessment of the distal radioulnar joint is routinely performed, intraoperative evaluation of residual instability after fracture fixation remains largely subjective and inconsistently documented. A standardized intraoperative grading system for distal radioulnar joint instability has been previously published and shown to have acceptable interobserver and intraobserver reliability [3]. Conceptually, this classification shares the advantage of flexibility with other widely used systems in wrist trauma, allowing application across different injury patterns and clinical contexts, including acute and chronic instability, as well as isolated or fracture-associated DRUJ injuries [4,5,6,7,8,9]. However, its clinical prognostic value has not been evaluated in a prospective multicenter setting.

The primary objective of the present study was to assess the prognostic validity of this intraoperative distal radioulnar joint instability grading system in patients with distal radius and Galeazzi fractures treated with plate fixation. We hypothesized that increasing instability severity would be independently associated with worse functional outcome at 12 months [10,11,12]. Secondary objectives were to examine the association between intraoperative instability grade and pain, grip strength, radiographic distal radioulnar joint gap, and postoperative complications [13,14,15,16]. To enhance external validity, this evaluation was conducted as a prospective multicenter study across two institutions with different healthcare systems, consistent with the design principles of multicenter clinical validation studies [17,18].

Distal radioulnar joint stability represents a continuum rather than a binary condition, reflecting progressive failure of the soft tissue and secondary stabilizing structures of the distal forearm. Partial insufficiency of the triangular fibrocartilage complex may be functionally compensated by secondary stabilizers such as the distal oblique bundle of the interosseous membrane and dynamic muscular restraints, whereas more advanced lesions are associated with combined failure of these structures and loss of joint congruence [19,20,21,22]. Experimental and clinical studies have demonstrated that disruption of these stabilizing systems alters load transfer and joint kinematics even after restoration of osseous alignment, supporting the concept that instability severity reflects the extent of underlying structural compromise rather than a purely radiographic finding [23,24,25]. An intraoperative grading system that captures increasing severity of instability therefore provides a biologically grounded framework for prognostic stratification.

## 2. Materials and Methods

### 2.1. Patient Selection

Patients were recruited between December 2021 and October 2024. Consecutive adult patients treated with plate fixation for distal radius fractures or Galeazzi fractures at participating centers were prospectively enrolled during the study period. Inclusion criteria were age ≥ 18 years, operative treatment with volar plate fixation, and availability of intraoperative distal radioulnar joint assessment and 12-month follow-up data. Exclusion criteria included prior ipsilateral wrist surgery, pathological fractures, open fractures requiring staged management, and incomplete follow-up.

### 2.2. Intraoperative Assessment of DRUJ Stability

**Figure 1:**
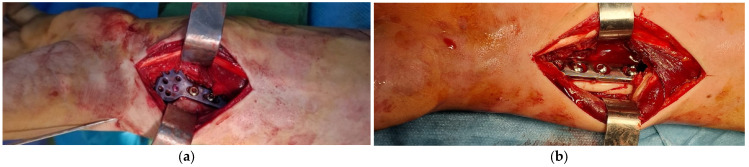
(**a**) Osteosynthesis of a distal radius fracture performed through a modified Henry approach using a titanium anatomical locking plate. (**b**) Osteosynthesis of a Galeazzi fracture performed through the Henry approach using a dynamic compression plate (DCP).

Fracture fixation was performed according to established orthopedic trauma principles in both centers, with the objective of restoring radial length, volar tilt, and radial inclination. Implant selection and fixation technique followed routine institutional practice and were determined by fracture morphology and intraoperative findings. This approach reflects real-world surgical practice and supports the robustness of the grading system across different technical environments (Figure 1) [8,26,27,28].

After fracture reduction and fixation [2,27,29], distal radioulnar joint stability was assessed intraoperatively by manual evaluation of ulnar head translation relative to the distal radius, considering both the magnitude of translation and the quality of the endpoint. Stability was further evaluated using reproducible maneuvers that have been thoroughly described in the literature, including anteroposterior stress testing, passive forearm rotation from pronation to supination with palpation of the ulnar head, and fluoroscopic assessment of radioulnar congruence [15,19,30,31,32,33]. All stability maneuvers were performed under anesthesia to eliminate muscular guarding and allow detection of subtle residual instability [26,34]. Intraoperative fluoroscopy was used to evaluate the DRUJ gap and to detect ulnar head subluxation on lateral views [16,35]. The presence of a palpable clunk or visible subluxation during dynamic testing was interpreted as residual instability [36,37,38].

Assessment was performed after definitive fracture fixation and before any additional DRUJ stabilizing procedure, ensuring that grading reflected residual instability rather than treatment effect. Instability was graded according to the criteria summarized in Table 1. Postoperative distal radioulnar joint stabilization and immobilization were not protocolized and were performed at the discretion of the treating surgeon based on intraoperative findings, introducing potential confounding by indication.

To ensure consistency of intraoperative grading across centers, all participating surgeons were familiarized with the grading system prior to study initiation. The classification had been previously published and its interobserver and intraobserver reliability had been evaluated in a separate validation study [3,39]. Before study enrollment, the grading criteria were reviewed locally, and surgeons applied the classification according to its predefined definitions during routine intraoperative assessment. The same assessment sequence, testing maneuvers, and fluoroscopic criteria were applied in both centers to ensure methodological consistency [16,38].

**Figure 2:**
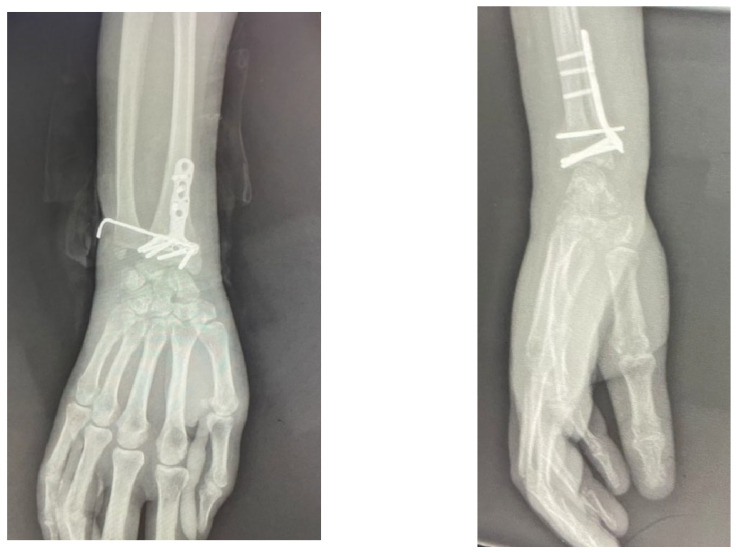
Osteosynthesis of a distal radius fracture associated with an ulnar styloid fracture using a polyaxial titanium locking plate (volar rim), with a Grade III distal radioulnar joint injury stabilized using a Kirschner wire.

In the present study, intraoperative grading was performed according to these predefined criteria using manual stress testing, dynamic forearm rotation, and fluoroscopic assessment. The intraoperative DRUJ grade was recorded as an observational variable and did not dictate fracture fixation strategy or postoperative management, which were determined by the treating surgeon according to fracture characteristics and overall intraoperative findings. In cases with intraoperative instability, additional stabilization procedures could be performed at the surgeon’s discretion, including temporary DRUJ transfixion with Kirschner wires, TFCC repair [40], and prolonged postoperative immobilization (Figure 2). Postoperative immobilization protocols and methods of distal radioulnar joint stabilization were not standardized and varied between centers, reflecting routine clinical practice and surgeon preference [17].

### 2.3. Outcome Measures

The primary outcome was functional disability at 12 months postoperatively, assessed using the QuickDASH questionnaire [41]. Secondary outcomes included pain intensity measured using a visual analog scale, grip strength assessed with a handheld dynamometer [13], radiographic evaluation of the distal radioulnar joint gap, and postoperative complications. The 12-month time point was selected to reflect stable functional recovery following fracture healing and rehabilitation [42].

Postoperative complications were recorded prospectively and categorized as any complication or major complications. Major complications were defined a priori as events with clear clinical or surgical relevance, including persistent distal radioulnar joint instability, post-traumatic arthritis, implant removal, deep infection, or reoperation. Isolated pain or stiffness without further intervention was not classified as a major complication.

Radiographic assessment included standard posteroanterior and lateral wrist radiographs, obtained with the forearm positioned in neutral rotation and the wrist in neutral flexion and extension. Ulnar variance, volar tilt, radial inclination, and distal radioulnar joint gap were measured using standard techniques [43,44,45]. For the present analysis, the distal radioulnar joint gap at 12 months was used as the primary radiographic correlate of intraoperative instability, because it reflects persistent soft tissue insufficiency rather than osseous alignment alone [46], while the remaining parameters were recorded to confirm acceptable fracture reduction and alignment [47,48].

### 2.4. Statistical Analysis

Statistical analyses were performed using IBM SPSS Statistics, version 27 (IBM Corp., Armonk, NY, USA). Descriptive statistics were used to summarize patient characteristics and outcomes according to intraoperative distal radioulnar joint status. Continuous variables were reported as means with standard deviations or medians with interquartile ranges, as appropriate, and categorical variables were reported as frequencies and percentages.

This study was designed as a prognostic cohort investigation rather than a comparative interventional trial. Intraoperative distal radioulnar joint instability grade was analyzed as an ordinal predictor reflecting increasing severity of instability, rather than as discrete comparison groups. Consequently, no a priori sample size calculation for between group comparisons was performed. The association between intraoperative instability severity and functional outcome was evaluated using multivariable linear regression, with QuickDASH score at 12 months as the dependent variable. Intraoperative DRUJ instability grade was modeled as an ordinal variable, with no DRUJ injury serving as the reference category. The model was adjusted for age, sex, fracture type, and center. Regression coefficients were reported with 95 percent confidence intervals and p values. Statistical significance was defined as a two-sided *p* value less than 0.05.

Because postoperative DRUJ stabilization and immobilization were not protocolized and could vary according to surgeon judgment, sensitivity analyses adjusting for postoperative stabilization and immobilization were performed to assess the robustness of the observed associations. These analyses were intended to support prognostic interpretation of the grading system under real-world conditions rather than to estimate causal effects of specific interventions.

## 3. Results

### 3.1. Study Population

A total of 120 patients with distal radius or Galeazzi fractures treated with plate fixation were included in the analysis. All patients completed clinical and radiological follow-up at 12 months. Baseline demographic and injury-related characteristics according to intraoperative distal radioulnar joint status are presented in Table 2. The distribution of age, sex, fracture type, and treatment center was comparable across groups.

### 3.2. Clinical and Radiological Outcomes at 12 Months

**Figure 3:**
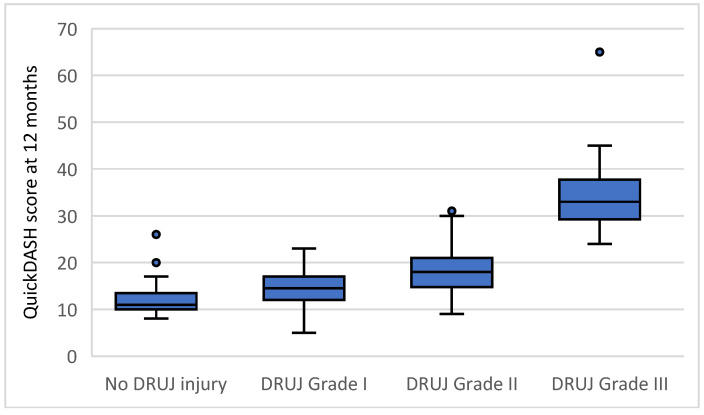
Distribution of QuickDASH scores at 12 months according to intraoperative distal radioulnar joint instability grade. Boxes represent the interquartile range, the horizontal line indicates the median, whiskers denote the range of non-outlier values, and points indicate outliers.

**Figure 4:**
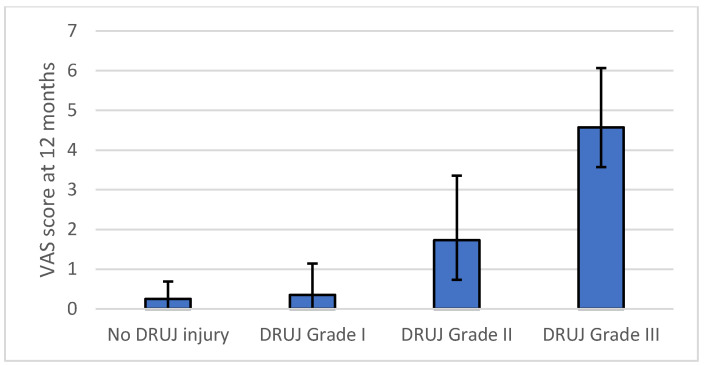
Pain at 12 months according to intraoperative distal radioulnar joint instability grade. Bars represent mean values, and error bars indicate dispersion.

**Figure 5:**
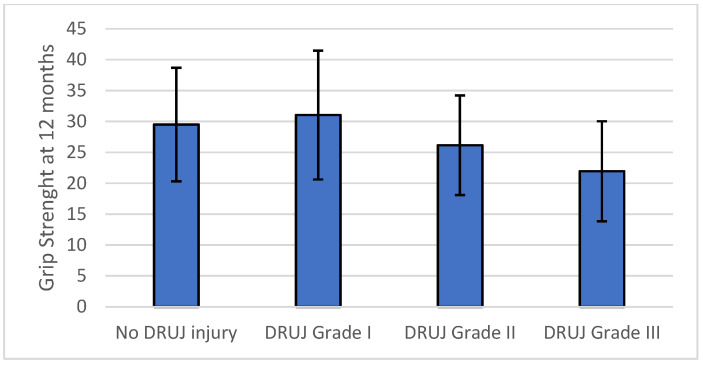
Grip strength at 12 months according to intraoperative distal radioulnar joint instability grade. Bars represent mean values, and error bars indicate dispersion.

Clinical and radiological outcomes according to intraoperative distal radioulnar joint status are summarized in Table 3. Mean QuickDASH scores increased progressively with higher grades of intraoperative instability, demonstrating a stepwise deterioration in patient-reported disability (Figure 3). Pain intensity assessed by visual analog scale (Figure 4) increased across instability grades, while grip strength (Figure 5) decreased with increasing instability severity.

**Figure 6:**
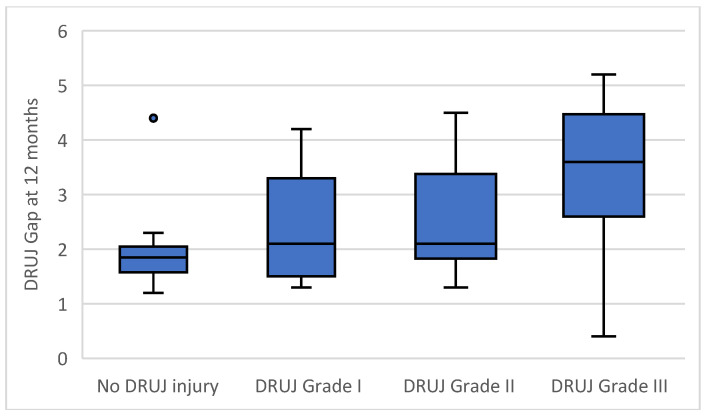
Distal radioulnar joint gap at 12 months according to intraoperative distal radioulnar joint instability grade. Boxes represent the interquartile range, the horizontal line indicates the median, whiskers denote the range of non-outlier values, and points indicate outliers.

Radiographic alignment parameters, including ulnar variance, volar tilt, and radial inclination, remained within acceptable ranges across all groups at 12 months, confirming satisfactory fracture reduction. In contrast, the distal radioulnar joint gap demonstrated a progressive increase with higher grades of intraoperative instability, reflecting residual joint incongruence rather than poor reduction in the fracture (Figure 6).

### 3.3. Multivariable Analyses

Multivariable linear regression analysis demonstrated that intraoperative distal radioulnar joint instability grade was independently associated with all evaluated clinical and radiological outcomes at 12 months, confirming the univariable trends observed in Table 3 and Figure 3, Figure 4, Figure 5 and Figure 6. For the primary outcome, each one-grade increase in instability severity was associated with a mean increase of 5.85 points in the QuickDASH score after adjustment for age, sex, fracture type, and treatment center (95 percent confidence interval 4.35 to 7.35, *p* < 0.001). None of the demographic or fracture-related variables were independently associated with QuickDASH score.

In secondary multivariable models, intraoperative instability grade remained the only independent predictor of postoperative distal radioulnar joint gap, with a mean increase of 0.52 mm per grade increase (95 percent confidence interval 0.318 to 0.729, *p* < 0.001). Higher instability grade was also independently associated with increased pain at 12 months, with a mean increase of 1.35 visual analog scale points per grade (95 percent confidence interval 1.08 to 1.63, *p* < 0.001), and with reduced grip strength, with a mean decrease of 3.23 kg per grade increase (95 percent confidence interval −4.62 to −1.85, *p* < 0.001). No multicollinearity was identified in any model, with variance inflation factors below 1.13 for all predictors. Multivariable regression results for all outcomes are summarized in Table 4.

In sensitivity analyses additionally adjusting the primary multivariable model for postoperative DRUJ stabilization and immobilization strategy, the association between intraoperative distal radioulnar joint instability grade and 12-month QuickDASH score remained unchanged. Each one-grade increase in instability severity was associated with a 5.9 point increase in QuickDASH score (*p* < 0.001) after accounting for postoperative management variables. Postoperative DRUJ stabilization was not independently associated with functional outcome (*p* = 0.58), and immobilization strategy did not demonstrate an independent effect on QuickDASH score.

### 3.4. Complications

Postoperative complications were more frequent in patients with higher grades of intraoperative instability, with major complications occurring predominantly in Grade II and Grade III.

Postoperative complications increased progressively with intraoperative instability severity (Table 2). The proportion of patients experiencing any complication rose from the stable group through Grades I and II, with the highest complication burden observed in patients with Grade III instability. Major complications were uncommon in patients without instability or with low-grade instability but occurred more frequently in higher grades. Owing to the limited number of patients with Grade III instability, estimates in this subgroup should be interpreted with caution.

### 3.5. Implant Type Analysis and Postoperative Immobilization

Functional outcomes and complication rates did not differ significantly according to implant type within each fracture category. Among patients with distal radius fractures, no statistically significant differences in QuickDASH score, pain intensity, grip strength, distal radioulnar joint gap, or complication rates were observed between volar locking plates and polyaxial constructs (all *p* > 0.05). Similarly, among patients with Galeazzi fractures, outcomes did not differ between dynamic compression plates and alternative fixation constructs (all *p* > 0.05). Implant type was therefore not included as an independent predictor in the multivariable regression models.

Postoperative immobilization strategy (sugar tong splint, long arm splint, or no immobilization) was not independently associated with functional or radiological outcomes at 12 months (all *p* > 0.05) and was therefore not included in the multivariable models.

## 4. Discussion

Previous research has largely focused on anatomical restoration or on the reliability of DRUJ assessment methods, without demonstrating a direct association between intraoperative instability severity and long-term functional outcomes [37,49,50]. By validating the grading system prospectively across different centers and treatment environments, the present study extends its utility beyond reproducibility and supports its role as a clinically relevant prognostic tool. In this cohort, increasing intraoperative instability severity was independently associated with worse functional and clinical outcomes at 12 months, including higher disability, greater pain, reduced grip strength, increased distal radioulnar joint widening, and a higher incidence of major postoperative complications [51].

These findings highlight that restoration of distal radius anatomy alone does not ensure functional recovery [24,52,53,54]. Although modern fixation techniques reliably correct osseous alignment, wrist biomechanics depend on the integrity of the distal radioulnar joint and its soft tissue stabilizers [25,55,56,57,58]. Residual instability may persist despite anatomically satisfactory fixation and represents a mechanism of postoperative dysfunction that is not captured by radiographic alignment parameters [43,59,60]. By evaluating instability after fracture stabilization, the present study specifically addresses residual joint behavior once bony factors have been corrected [61,62,63].

A key observation in this study is the graded relationship between intraoperative instability severity and outcome. Rather than a binary phenomenon, distal radioulnar joint instability demonstrated a proportional association with disability, pain, and objective functional impairment [51]. The stepwise increase in QuickDASH scores, parallel deterioration in grip strength, and progressive widening of the distal radioulnar joint gap support the construct validity of the grading system as a measure of residual joint dysfunction with prognostic significance [35,64].

This graded relationship is biologically plausible when considered in the context of distal forearm stabilizer anatomy and biomechanics. Lower grades of instability likely reflect isolated or partial insufficiency of the triangular fibrocartilage complex, with preserved function of secondary stabilizers such as the distal oblique bundle of the interosseous membrane and dynamic muscular restraints [19,20,21,22]. In contrast, higher grades of instability may indicate combined failure of the triangular fibrocartilage complex, capsuloligamentous restraints, and interosseous membrane, resulting in persistent joint incongruence, abnormal load transmission, and progressive functional impairment despite restoration of osseous alignment [25,55,65,66]. Biomechanical studies have demonstrated that combined disruption of these stabilizing systems leads to significantly increased ulnar head translation and altered joint kinematics during pronosupination, providing a mechanistic explanation for the worse clinical outcomes observed at higher instability grades [67,68,69].

Although intraoperative fluoroscopy was used as part of the stability assessment, the postoperative distal radioulnar joint gap represents a distinct radiographic outcome measured under standardized conditions at 12 months. Intraoperative evaluation was qualitative and dynamic, aimed at detecting residual instability after fracture fixation, whereas postoperative radiographic measurements reflected static joint alignment after biological healing and functional loading [35,59,70].

The observed radiographic findings further reinforce the clinical relevance of the intraoperative assessment. While ulnar variance, volar tilt, and radial inclination were restored across all groups, the distal radioulnar joint gap increased proportionally with instability grade and was independently predicted by intraoperative instability severity [42,71,72,73]. This suggests that the DRUJ gap may represent a structural correlate of soft tissue insufficiency, whereas static alignment parameters alone are insufficient to characterize postoperative joint function [15,30].

The prognostic value demonstrated in this study extends previous work establishing the reproducibility of the grading system. While reliability is a prerequisite for clinical adoption, prognostic validity confirms that the classification captures information directly relevant to patient outcomes [16,51,74,75]. Together, these findings support the grading system as a clinically meaningful descriptor rather than a purely descriptive or academic construct.

Conceptually, this supports the interpretation of distal radioulnar joint instability as a continuum of structural failure rather than a dichotomous condition. By integrating clinical testing, dynamic assessment, and fluoroscopic evaluation into a single ordinal framework, the grading system captures progressive loss of stabilizer integrity that is not represented in existing fracture classifications or static radiographic parameters [7,20,76,77]. This feature may explain its ability to stratify postoperative risk despite heterogeneity in fracture pattern, implant choice, and immobilization strategy.

This study has several strengths. The prospective multicenter design enhances external validity, and consistent associations across two centers support generalizability across different clinical environments. Adjustment for center and demographic variables further reduces the influence of local practice patterns. Importantly, intraoperative grading was recorded observationally and did not dictate management, allowing evaluation of prognostic value independent of treatment strategy [18].

Although intraoperative distal radioulnar joint stabilization was not protocolized, differences in practice between centers reflect real-world variability [1,72,78]. In Romania, temporary Kirschner wire transfixion was used more frequently, whereas in Jordan, TFCC repair and modern implants were applied more often [40,79]. Importantly, the absence of significant differences in functional outcomes between centers supports the interpretation that biomechanical principles and appropriate intraoperative stability assessment may outweigh variations in implant availability or local preferences [28,80]. The absence of a measurable effect of postoperative immobilization strategy on outcomes suggests that intraoperative stabilization and residual joint mechanics may have a greater influence on recovery than immobilization type itself.

The present findings suggest that the intraoperative instability grade identifies a subgroup of patients at increased risk of persistent dysfunction, in whom the threshold for additional stabilization may justify closer consideration of additional stabilization [78,81]. While the current study was not designed to compare stabilization strategies, the results support the role of the grading system as a framework for future interventional studies evaluating grade-specific management algorithms.

### Limitations

Several limitations must be acknowledged. Intraoperative assessment relies on clinical judgment and therefore involves inherent subjectivity, although this reflects routine surgical practice. Postoperative distal radioulnar joint stabilization and immobilization were not protocolized and were performed at the discretion of the treating surgeon based on intraoperative findings, introducing potential confounding by indication. Although sensitivity analyses were performed to evaluate the robustness of the association between instability grade and outcome, residual confounding cannot be excluded.

The study was not designed to evaluate specific treatment strategies based on instability grade, and the number of patients with Grade III instability was limited, requiring cautious interpretation of complication rates in this subgroup. Radiological assessment was limited to standard imaging, and dynamic modalities were not employed [82,83]. The presence and morphology of associated ulnar styloid fractures were not systematically recorded and therefore could not be analyzed as potential modifiers of distal radioulnar joint instability or functional outcome [84,85]. Finally, longer-term follow-up may be required to evaluate the relationship between residual instability and degenerative changes [14,29,60].

Because the grading system was developed by the same investigative group, observer familiarity may have influenced intraoperative assessment despite prior reliability validation, and independent external validation remains necessary. Future randomized or protocol-driven studies are required to determine whether grade-specific stabilization strategies can modify postoperative outcomes [17,86]. Intraoperative assessment of distal radioulnar joint stability relied on clinical judgment and manual testing and therefore involved inherent subjectivity. Instability grading was based on qualitative intraoperative evaluation performed by surgeons aware of the study’s aims. Although prior interobserver reliability of the grading system has been reported, the absence of blinding and the subjective nature of assessment represent important sources of potential bias. The multivariable models included a limited set of covariates, and potentially relevant confounders such as bone quality, fracture comminution, surgeon experience, and postoperative rehabilitation protocols could not be accounted for in the analysis [36,87,88]. The consistent prognostic associations observed in this study support future development of standardized intraoperative measurement methods to improve the reproducibility of instability grading [84,85].

In summary, systematic intraoperative assessment of distal radioulnar joint instability provides prognostic information not captured by fracture characteristics or radiographic alignment alone. The consistent association between intraoperative instability grade and outcome across centers supports external validity and indicates that the grading system can stratify risk in routine clinical practice, providing a foundation for future interventional studies evaluating grade-specific management strategies [1,18,89].

Although advanced imaging modalities and biomechanical assessments can further characterize stabilizer integrity and joint kinematics, the present study intentionally focused on intraoperative clinical assessment to reflect the information available to the surgeon at the time of decision making, consistent with previous clinical and biomechanical frameworks of DRUJ evaluation [67,90].

## 5. Conclusions

Intraoperative distal radioulnar joint instability severity assessed after fracture reduction and fixation is independently associated with functional, clinical, and radiological outcomes at 12 months in patients with distal radius and Galeazzi fractures treated with plate fixation. Increasing instability grades correspond to progressively worse disability, higher pain, reduced grip strength, greater distal radioulnar joint widening, and higher complication rates. 

These findings provide clinical validation of the previously proposed intraoperative distal radioulnar joint instability classification by demonstrating its prognostic relevance in a multicenter cohort. The classification functions as a practical and reproducible tool for stratifying residual joint dysfunction following anatomically successful fracture fixation. Its use may facilitate improved postoperative risk stratification and provides a foundation for future studies evaluating targeted management strategies in patients with higher grades of residual instability.

Beyond its prognostic value, the grading system offers a clinically intuitive framework that may assist intraoperative decision making regarding the need for additional DRUJ stabilization.

While causality cannot be inferred from the observational design, the consistency of associations across outcomes and centers supports the prognostic value of intraoperative instability grading in routine clinical practice.

## Figures and Tables

**Table 1:** Summary of distal radioulnar joint instability grades as originally defined.

Grade	Instability Severity	Joint Alignment	Dynamic Behavior
Grade I	Low-grade instability	Joint alignment preserved	Increased laxity with preserved stability throughout forearm rotation
Grade II	Moderate instability	Dynamic or transient subluxation	Reproducible instability or palpable clunk during forearm rotation
Grade III	High-grade instability	Fixed or easily reproducible dislocation	Gross instability with loss of functional joint congruence

**Table 2:** Baseline characteristics according to intraoperative distal radioulnar joint status.

Variable	No DRUJ Injury (*n* = 20)	DRUJ Grade I (*n* = 46)	DRUJ Grade II (*n* = 40)	DRUJ Grade III (*n* = 14)	Total (*n* = 120)
Age, years, mean ± SD	49.9 ± 13.7	48.5 ± 14.4	51.3 ± 12.8	50.9 ± 12.6	50.0 ± 13.5
Female, *n* (%)	15 (75.0)	25 (54.3)	22 (55.0)	7 (50.0)	69 (57.5)
Right side, *n* (%)	10 (50.0)	25 (54.3)	23 (57.5)	6 (42.9)	64 (53.3)
Distal radius fracture, *n* (%)	8 (40.0)	23 (50.0)	19 (47.5)	10 (71.4)	60 (50.0)
Romania center, *n* (%)	9 (45.0)	23 (50.0)	22 (55.0)	6 (42.9)	60 (50.0)

Instability grading refers to the proposed classification Grades I to III. Cases with no instability after fracture reduction and fixation were classified as no DRUJ injury and used as the reference category for analyses.

**Table 3:** Clinical and radiological outcomes at 12 months according to intraoperative DRUJ status.

Outcome	No DRUJ Injury (*n* = 20)	DRUJ Grade I (*n* = 46)	DRUJ Grade II (*n* = 40)	DRUJ Grade III (*n* = 14)
QuickDASH 12 months, mean ± SD	12.70 ± 4.80	14.17 ± 4.56	17.12 ± 6.60	35.14 ± 10.66
VAS 12 months, mean ± SD	0.25 ± 0.44	0.35 ± 0.79	1.73 ± 1.63	4.57 ± 1.50
Grip strength 12 months (kg), mean ± SD	29.50 ± 9.19	31.04 ± 10.43	26.15 ± 8.07	21.93 ± 8.10
DRUJ gap (mm), mean ± SD	1.85 ± 0.70	1.54 ± 0.68	2.27 ± 1.05	3.38 ± 1.51
Any complication, *n* (%)	2 (10.0)	9 (19.6)	24 (60.0)	14 (100.0)
Major complications, *n* (%)	0 (0.0)	3 (6.5)	11 (27.5)	9 (64.3)

**Table 4:** Multivariable linear regression models for 12-month outcomes.

Predictor	QuickDASH β (95% CI)	*p* Value	VAS β (95% CI)	*p* Value	Grip Strength β (95% CI)	*p* Value	DRUJ Gap β (95% CI)	*p* Value
DRUJ instability grade (per grade increase)	5.85 (4.35 to 7.35)	< 0.001	1.35 (1.08 to 1.63)	<0.001	−3.23 (−4.62 to −1.85)	<0.001	0.52 (0.32 to 0.73)	<0.001
Age (per year)	0.09 (−0.02 to 0.19)	0.107	0.01 (−0.01 to 0.03)	0.559	−0.37 (−0.47 to −0.28)	<0.001	0.00 (−0.01 to 0.02)	0.562
Sex (male vs. female)	0.75 (−2.02 to 3.53)	0.592	−0.49 (−0.99 to 0.02)	0.057	10.91 (8.36 to 13.47)	<0.001	−0.14 (−0.52 to 0.24)	0.459
Fracture type (Galeazzi vs. distal radius)	−0.82 (−3.49 to 1.85)	0.544	−0.28 (−0.76 to 0.21)	0.260	1.48 (−0.98 to 3.95)	0.236	−0.01 (−0.38 to 0.36)	0.955
Center (Jordan vs. Romania)	2.22 (−0.57 to 5.01)	0.118	−0.06 (−0.56 to 0.45)	0.825	−2.62 (−5.20 to −0.05)	0.046	0.21 (−0.18 to 0.59)	0.284

Each outcome was analyzed using a separate multivariable linear regression model adjusted for age, sex, fracture type, and treatment center. DRUJ instability grade was modeled as an ordinal variable, with increasing instability severity modeled as an ordinal variable. β coefficients represent the mean change in outcome per one-grade increase in instability severity. Positive β values indicate worse outcomes for QuickDASH, VAS, and DRUJ gap, and lower grip strength.

## Data Availability

The data are not publicly available due to institutional regulations and patient privacy restrictions but are available from the corresponding authors upon reasonable request.

## References

[1] Xiao A.X., Graf A.R., Dawes A., Daley C., Wagner E.R., Gottschalk M.B. (2021). Management of Acute Distal Radioulnar Joint Instability Following a Distal Radius Fracture: A Systematic Review and Meta-Analysis. J. Hand Surg. Glob. Online.

[2] Garg R., Mudgal C. (2020). Galeazzi Injuries. Hand Clin..

[3] Dmour A., Tirnovanu S.D., Popescu D.C., Forna N., Pinteala T., Dmour B.A., Savin L., Veliceasa B., Filip A., Carp A.C. (2024). Advancements in Diagnosis and Management of Distal Radioulnar Joint Instability: A Comprehensive Review Including a New Classification for DRUJ Injuries. J. Pers. Med..

[4] Kramer S.B., Raad F., Hauser A., Schipper I.B., Schep N.W.L. (2025). The Transverse Sigmoid Notch Morphology Unravelled. J. Hand Surg. Asian Pac. Vol..

[5] Atzei A., Luchetti R. (2011). Foveal TFCC tear classification and treatment. Hand Clin..

[6] Schmitt R., Kunz A.S., Reidler P., Huflage H., Hesse N. (2024). Triangular Fibrocartilage Complex (TFCC)—Anatomy, Imaging, and Classifications with Special Focus on the CUP Classification. RöFo—Fortschritte Geb. Röntgenstrahlen Bildgeb. Verfahr..

[7] Rettig M.E., Raskin K.B. (2001). Galeazzi fracture-dislocation: A new treatment-oriented classification. J. Hand Surg. Am..

[8] Jayakumar P., Teunis T., Giménez B., Verstreken F., Di Mascio L., Jupiter J. (2016). AO Distal Radius Fracture Classification: Global Perspective on Observer Agreement. J. Wrist Surg..

[9] Illarramendi A., González Della Valle A., Segal E., De Carli P., Maignon G., Gallucci G. (1998). Evaluation of simplified Frykman and AO classifications of fractures of the distal radius. Int. Orthop..

[10] Kwan S.A., McEntee R., Sodha S., Kwok M., Beredjiklian P.K., Tulipan J.E. (2025). Outcomes in Patients with Bilateral Distal Radius Fractures. J. Wrist Surg..

[11] Zong S.L., Kan S.L., Su L.X., Wang B. (2015). Meta-analysis for dorsally displaced distal radius fracture fixation: Volar locking plate versus percutaneous Kirschner wires. J. Orthop. Surg. Res..

[12] Holmqvist K.J., Johnson T., Fornander L. (2024). The Choice of Osteosynthesis for Distal Radius Fractures: A Matter of Taste, Fracture Instability, or Patient-Related Factors? A Retrospective Study of Functional Outcome in 346 Distal Radius Fractures Operated with Percutaneous Wires or Volar Plate Fixation. HAND.

[13] Lee S.H., Gong H.S. (2022). Grip Strength Measurement for Outcome Assessment in Common Hand Surgeries. Clin. Orthop. Surg..

[14] Giddins G. (2023). The distal radioulnar joint after distal radial fractures: When and how do we need to treat pain, stiffness or instability?. J. Hand Surg..

[15] Giddins G., Fraser T., Lambert R. (2025). Subacute distal radio-ulnar joint subluxation after a distal radius fracture. J. Hand Surg..

[16] Meaike J.D., Meaike J.J., Amrami K.K., Kakar S. (2024). Validating Clinical Distal Radioulnar Joint Examination with Radiographic Parameters. HAND.

[17] Costa M.L., Achten J., Ooms A., Png M.E., Cook J.A., Lamb S.E., Hedley H., Dias J. (2022). Surgical fixation with K-wires versus casting in adults with fracture of distal radius: DRAFFT2 multicentre randomised clinical trial. BMJ.

[18] Perry D.C., Achten J., Mason J., Kounail D., Nicolaou N., Metcalfe D., Lyttle M., Tutton E., Appelbe D., Gibson P. (2025). The protocol for a multicentre prospective randomized noninferiority trial of surgical reduction versus non-surgical casting for displaced distal radius fractures in children. Bone Jt. Open.

[19] Quadlbauer S., Pezzei C., Hintringer W., Hausner T., Leixnering M. (2018). Clinical examination of the distal radioulnar joint. Orthopade.

[20] Low S.L., Clippinger B.B., Landfair G.L., Criner-Woozley K. (2020). A Biomechanical Evaluation of the DRUJ After Distal Oblique Bundle Reconstruction. J. Hand Surg..

[21] Yoshii Y., Yuine H., Tung W., Ishii T. (2019). Quantitative assessment of distal radioulnar joint stability with pressure-monitor ultrasonography. J. Orthop. Surg. Res..

[22] Yin S., Zhang C., Huang Y., Pan J., Wang X., Liu X. (2025). Comparative study regarding the stability of a proximal ulnar stump with or without distal oblique bundle reconstruction during the Sauvé–Kapandji procedure: A finite-element analysis. Front. Bioeng. Biotechnol..

[23] Faucher G.K., Zimmerman R.M., Zimmerman N.B. (2016). Instability and arthritis of the distal radioulnar joint: A critical analysis review. JBJS Rev..

[24] Huang J.I., Hanel D.P. (2012). Anatomy and Biomechanics of the Distal Radioulnar Joint. Hand Clin..

[25] Orbay J.L., Cambo R.A. (2020). Biomechanical Factors in Stability of the Forearm. Hand Clin..

[26] Wolfe S.W., Pederson W.C., Kozin S.H., Cohen M.S. (2021). Green’s Operative Hand Surgery.

[27] Tornetta P., Ricci W.M., Ostrum R.F., McQueen M.M., McKee M.D., Court-Brown C.M. (2019). Rockwood and Green’s Fractures in Adults.

[28] Goldfarb C. (2004). Hand Surgery, Volumes I and II. J. Hand Surg. Am..

[29] Ermutlu C., Mert M., Kovalak E., Kanay E., Obut A., Öztürkmen Y. (2020). Management of Distal Radius Fractures: Comparison of Three Methods. Cureus.

[30] Iida A., Omokawa S., Akahane M., Kawamura K., Takayama K., Tanaka Y. (2012). Distal Radioulnar Joint Stress Radiography for Detecting Radioulnar Ligament Injury. J. Hand Surg. Am..

[31] Flores D.V., Umpire D.F., Rakhra K.S., Jibri Z., Belmar G.A.S. (2023). Distal Radioulnar Joint: Normal Anatomy, Imaging of Common Disorders, and Injury Classification. RadioGraphics.

[32] Mirghasemi A.R., Lee D.J., Rahimi N., Rashidinia S., Elfar J.C. (2015). Distal Radioulnar Joint Instability. Geriatr. Orthop. Surg. Rehabilit..

[33] Nagashima M., Omokawa S., Hasegawa H., Nakanishi Y., Kawamura K., Tanaka Y. (2024). Reliability and Validity Analysis of the Distal Radioulnar Joint Ballottement Test. J. Hand Surg. Am..

[34] Stuart P.R., Berger R.A., Linscheid R.L., An K.N. (2000). The dorsopalmar stability of the distal radioulnar joint. J. Hand Surg. Am..

[35] Werner F., LeVasseur M., Harley B., Anderson A. (2016). Role of the Interosseous Membrane in Preventing Distal Radioulnar Gapping. J. Wrist Surg..

[36] Wijffels M., Brink P., Schipper I. (2012). Clinical and Non-Clinical Aspects of Distal Radioulnar Joint Instability. Open Orthop. J..

[37] Pickering G.T., Fine N.F., Knapper T.D., Giddins G.E.B. (2022). The reliability of clinical assessment of distal radioulnar joint instability. J. Hand Surg..

[38] Giddins G., Knapper T., Fine N., Pickering G. (2024). The reliability of clinical assessment of distal radioulnar joint instability among non-United Kingdom European surgeons. J. Hand Surg..

[39] Dmour A., Burlui A.M., Dmour B.-A., Tîrnovanu Ș.-D., Popescu D.-C., Tîrnovanu M.C., Savin L., Pertea M., Cozma T., Carp A.C. (2026). Interobserver and Intraobserver Reliability of a Novel Classification System for Distal Radioulnar Joint Instability. Life.

[40] Khair Y., Mustafa A., Mestrihi S., Azzam E., Al-Qasaimeh M., Awad D., Ovidiu A. (2023). Outcome in TFCC repair using micro anchor and trans-osseous technique. Exp. Ther. Med..

[41] Galardini L., Coppari A., Pellicciari L., Ugolini A., Piscitelli D., La Porta F., Bravini E., Vercelli S. (2024). Minimal Clinically Important Difference of the Disabilities of the Arm, Shoulder and Hand (DASH) and the Shortened Version of the DASH (QuickDASH) in People with Musculoskeletal Disorders: A Systematic Review and Meta-Analysis. Phys. Ther..

[42] Hall M.J., Ostergaard P.J., Rozental T.D. (2021). Outcome Measurement for Distal Radius Fractures. Hand Clin..

[43] Takemoto R., Sugi M., Immerman I., Tejwani N., Egol K.A. (2014). Ulnar variance as a predictor of persistent instability following Galeazzi fracture-dislocations. J. Orthop. Traumatol..

[44] Heiss-Dunlop W., Couzens G.B., Peters S.E., Gadd K., Di Mascio L., Ross M. (2014). Comparison of Plain X-Rays and Computed Tomography for Assessing Distal Radioulnar Joint Inclination. J. Hand Surg. Am..

[45] Ng A.W., Tong C.S., Hung E.H., Griffith J.F., Tse W., Wong C.W., Mak M.C., Ho P. (2019). Top-Ten Tips for Imaging the Triangular Fibrocartilaginous Complex. Semin. Musculoskelet. Radiol..

[46] Ward L.D., Ambrose C.G., Masson M.V., Levaro F. (2000). The role of the distal radioulnar ligaments, interosseous membrane, and joint capsule in distal radioulnar joint stability. J. Hand Surg. Am..

[47] Squires J.H., England E., Mehta K., Wissman R.D. (2014). The role of imaging in diagnosing diseases of the distal radioulnar joint, triangular fibrocartilage complex, and distal ulna. Am. J. Roentgenol.

[48] Amrami K.K., Moran S.L., Berger R.A., Ehman E.C., Felmlee J.P. (2010). Imaging the distal radioulnar joint. Hand Clin..

[49] D’Sa H., Willing R., Murray T., Rowan K., Grewal R., King G., Daneshvar P. (2023). Reliability of the Sigmoid Notch Classification of the Distal Radioulnar Joint. J. Wrist Surg..

[50] Sharma M., Choudhury S.R., Soundararajan R., Sheth R., Sinha A., Prakash M. (2024). Reliability of distal radius fracture classification systems: A CT based study. Emerg. Radiol..

[51] Birajdar A., Kumar S., Phalak M., Chaudhari T., Meghana D. (2025). Comprehensive Management Approaches for Acute Distal Radioulnar Joint Instability Post distal End Radius Fracture. J. Orthop. Case Rep..

[52] Nypaver C., Bozentka D.J. (2021). Distal Radius Fracture and the Distal Radioulnar Joint. Hand Clin..

[53] du Plessis P., Fournier M.C. (2023). Management of complex wrist fractures with volar and dorsal locked (double-locked) K-lock. OTA Int. Open Access J. Orthop. Trauma.

[54] Lee H.W., Kim K.T., Lee S., Yoon J.H., Kim J.Y. (2024). Fracture Severity and Triangular Fibrocartilage Complex Injury in Distal Radius Fractures with or without Osteoporosis. J. Clin. Med..

[55] Eschweiler J., Li J., Quack V., Rath B., Baroncini A., Hildebrand F., Migliorini F. (2022). Anatomy, Biomechanics, and Loads of the Wrist Joint. Life.

[56] Bunch P.M., Sheehan S.E., Dyer G.S., Sodickson A., Khurana B. (2016). A biomechanical approach to distal radius fractures for the emergency radiologist. Emerg. Radiol..

[57] Dmour A., Toma Ș.-L., Cazac A.-M., Tirnovanu S.D., Dima N., Dmour B.-A., Popescu D.C., Alexa O. (2024). Comparative Biomechanical Analysis of Kirschner Wire Fixation in Dorsally Displaced Distal Radius Fractures. Life.

[58] Shen J., Papadonikolakis A., Garrett J.P., Davis S.M., Ruch D.S. (2005). Ulnar-Positive Variance as a Predictor of Distal Radioulnar Joint Ligament Disruption. J. Hand Surg. Am..

[59] Poppler L.H., Moran S.L. (2020). Acute Distal Radioulnar Joint Instability: Evaluation and Treatment. Hand Clin..

[60] Spies C.K., Langer M., Müller L.P., Oppermann J., Unglaub F. (2020). Distal radioulnar joint instability: Current concepts of treatment. Arch. Orthop. Trauma Surg..

[61] Marcheix P.-S., Delclaux S., Ehlinger M., Scheibling B., Dalmay F., Hardy J., Bonnevialle P. (2024). Determinants of non-union after standard plate fixation for combined radial and ulnar fractures in adults. Injury.

[62] Wijffels M., Ring D. (2016). The Influence of Non-union of the Ulnar Styloid on Pain, Wrist Function and Instability after Distal Radius Fracture. J. Hand Microsurg..

[63] Kolovich G.P., Heifner J.J., Falgiano P.A., Mahoney B. (2024). Distal Radioulnar Joint Instability. J. Orthop. Trauma.

[64] Peña-Martínez V.M., Villanueva-Guerra E., Tamez-Mata Y., Simental-Mendía M., Gallardo-Madrid A., Blázquez-Saldaña J., Acosta-Olivo C. (2024). Distal radius fractures: Classifications concordance among orthopedic residents on a teaching hospital. J. Orthop. Sci..

[65] Moritomo H. (2012). The distal interosseous membrane: Current concepts in wrist anatomy and biomechanics. J. Hand Surg..

[66] Angelis S., Apergis E., Apostolopoulos A., Vlasis K., Piagkou M., Filippou D., Kanellos P.D. (2023). The Distal Oblique Bundle in the Distal Forearm: From Anatomical Features to Clinical Implementation. Cureus.

[67] Oldfield C.E., Boland M.R., Greybe D., Hing W. (2017). Ultrasound imaging of the distal radioulnar joint: A new method to assess ulnar radial translation in forearm rotation. J. Hand Surg..

[68] Hojo J., Omokawa S., Iida A., Ono H., Moritomo H., Tanaka Y. (2019). Three-Dimensional Kinematic Analysis of the Distal Radioulnar Joint in the Axial-Loaded Extended Wrist Position. J. Hand Surg..

[69] Sanders L., Johnson N., Dias J.J. (2022). Kirschner Wire Fixation in Dorsally Displaced Distal Radius Fractures: A Biomechanical Evaluation. J. Wrist Surg..

[70] Carlsen B.T., Dennison D.G., Moran S.L. (2010). Acute Dislocations of the Distal Radioulnar Joint and Distal Ulna Fractures. Hand Clin..

[71] Hruby L.A., Haider T., Laggner R., Gahleitner C., Erhart J., Stoik W., Hajdu S., Thalhammer G. (2022). Standard radiographic assessments of distal radius fractures miss involvement of the distal radioulnar joint: A diagnostic study. Arch. Orthop. Trauma Surg..

[72] Li C., Kong L., Shi X., Zhang Z., Lu J., Zhang B. (2023). Predictive factors of distal radioulnar joint instability after surgical treatment of distal radius fractures. Medicine.

[73] Trehan S.K., Orbay J.L., Wolfe S.W. (2015). Coronal shift of distal radius fractures: Influence of the distal interosseous membrane on distal radioulnar joint instability. J. Hand Surg..

[74] Oh C., Fort M.W., Kakar S. (2024). Validation of the Clenched Fist View in Detecting Scapholunate Ligamentous Injury. HAND.

[75] Verhiel S.H.W.L., Özkan S., Langhammer C.G., Chen N.C. (2020). The Serially-Operated Essex-Lopresti Injury: Long-Term Outcomes in a Retrospective Cohort. J. Hand Microsurg..

[76] Fontana M., Rotini M., Battiston B., Artiaco S., Dutto E., Sard A., Colozza A., Vicenti G., Cavallo M., Rotini R. (2023). Unstable lesions of the forearm: Terminology, evaluative score and synoptic table. Injury.

[77] Kakar S., Garcia-Elias M. (2016). The “Four-Leaf Clover” Treatment Algorithm: A Practical Approach to Manage Disorders of the Distal Radioulnar Joint. J. Hand Surg. Am..

[78] Zampetakis K., Stavrakakis I.M., Alpantaki K., Kastanis G., Ktistakis I., Tsioupros A., Ritzakis N., Chaniotakis C. (2024). Systematic Review of Acute Isolated Distal Radioulnar Joint Dislocation: Treatment Options. J. Clin. Med..

[79] Mccarron L., Coombes B.K., Bindra R., Bisset L. (2024). Post-surgical Rehabilitation Guidelines for Triangular Fibrocartilage Complex Foveal Repair: A Survey of Australian Hand and Wrist Surgeons. J. Hand Surg. Asian Pac. Vol..

[80] Lo I.N., Chen K.J., Yin C.Y., Huang H.K., Wang J.P., Huang Y.C. (2024). Comparing the Outcomes of Suture Anchor Repair and Rein-Type Capsular Suture for Triangular Fibrocartilage Complex Foveal Tears with a Minimum 2-Year Follow-Up. J. Hand Surg..

[81] Qazi S., Graham D., Regal S., Tang P., Hammarstedt J.E. (2021). Distal Radioulnar Joint Instability and Associated Injuries: A Literature Review. J. Hand Microsurg..

[82] Braig Z.V., Dittman L.E., Amrami K.K., Kakar S. (2024). Dynamic Computed Tomography of the Distal Radioulnar Joint Versus Magnetic Resonance Imaging in Detecting Foveal Tears of the Triangular Fibrocartilage Complex. HAND.

[83] Zhao X., Yu A., Zhao H., Qiu Y. (2024). Diagnostic value of MRI in traumatic triangular fibrocartilage complex injuries: A retrospective study. BMC Musculoskelet. Disord..

[84] Chen A.C.Y., Chiu C.H., Weng C.J., Chang S.S., Cheng C.Y. (2018). Early and late fixation of ulnar styloid base fractures yields different outcomes. J. Orthop. Surg. Res..

[85] Wong K.C., Wu M.W.F., Zai Q.J.J., Wong M.K., Howe T.S., Koh S.B.J., Soeharno H. (2023). Concomitant Ulnar Styloid Fractures in Distal Radius Osteosynthesis Does Not Impact Radiographic Outcomes, Ulnar Sided Symptoms and Patient Outcomes. Malays. Orthop. J..

[86] Kaivorinne A., Räisänen M.P., Karjalainen T., Jokihaara J., Gvozdenovic R., Wilcke M., Reito A., Anttila T., Pönkkö A., Lauridsen C. (2024). tREatment of trIaNgular FibrOcaRtilage ComplEx Ruptures (REINFORCER): Protocol for randomised, controlled, blinded, efficacy trial of triangular fibrocartilage complex tears. BMJ Open.

[87] Ajit Singh V., Jia T.Y., Devi Santharalinggam R., Gunasagaran J. (2023). Relationship of ulna styloid fracture to the distal radio-ulnar joint stability. A clinical, functional, and radiographic outcome study. PLoS ONE.

[88] Schmauss D., Pöhlmann S., Lohmeyer J.A., Germann G., Bickert B., Megerle K. (2016). Clinical tests and magnetic resonance imaging have limited diagnostic value for triangular fibrocartilaginous complex lesions. Arch. Orthop. Trauma Surg..

[89] Logan A.J., Lindau T.R. (2008). The management of distal ulnar fractures in adults: A review of the literature and recommendations for treatment. Strateg. Trauma Limb Reconstr..

[90] Adams B.D. (2000). Anatomic reconstruction of the distal radioulnar ligaments for DRUJ instability. Tech. Hand Up. Extrem. Surg..

